# Professionals’ perspectives on factors within primary mental health services that can affect pathways to involuntary psychiatric admissions

**DOI:** 10.1186/s13033-020-00417-z

**Published:** 2020-11-19

**Authors:** Irene Wormdahl, Tonje Lossius Husum, Jorun Rugkåsa, Marit B. Rise

**Affiliations:** 1 NTNU Social Research, Norwegian Resource Centre for Community Mental Health, 7491 Trondheim, Norway; 2 Department of Mental Health, Faculty of Medicine and Health Sciences, Norwegian University of Science and Technology, Trondheim, Norway; 3 Centre for Medical Ethics, Institute for Health & Society, University of Oslo, Oslo, Norway; 4 Health Service Research Unit, Akershus University Hospital, Lørenskog, Norway; 5 Centre for Care Research, University of South-Eastern Norway, Notodden, Norway

**Keywords:** Primary mental health care, Mental health services, Involuntary admission, Mental health recovery, Mental health, Psychiatry

## Abstract

**Background:**

Reducing involuntary psychiatric admissions has been on the international human rights and health policy agenda for years. Despite the last decades’ shift towards more services for adults with severe mental illness being provided in the community, most research on how to reduce involuntary admissions has been conducted at secondary health care level. Research from the primary health care level is largely lacking. The aim of this study was to explore mental health professionals’ experiences with factors within primary mental health services that might increase the risk of involuntary psychiatric admissions of adults, and their views on how such admissions might be avoided.

**Methods:**

Qualitative semi-structured interviews with thirty-two mental health professionals from five Norwegian municipalities. Data were analysed according to the Systematic Text Condensation method.

**Results:**

Within primary mental health care professionals experienced that a number of factors could increase the risk of involuntary psychiatric admissions. Insufficient time and flexibility in long-term follow-up, limited resources, none or arbitrary use of crisis plans, lack of tailored housing, few employment opportunities, little diversity in activities offered, limited opportunities for voluntary admissions, inadequate collaboration between services and lack of competence were some of the factors mentioned to increase the risk of involuntary psychiatric admissions. Several suggestions on how involuntary psychiatric admissions might be avoided were put forward.

**Conclusions:**

Mental health professionals within primary mental health care experienced that their services might play an active part in preventing the use of involuntary psychiatric admissions, suggesting potential to facilitate a reduction by intervening at this service level. Health authorities’ incentives to reduce involuntary psychiatric admissions should to a greater extent incorporate the primary health care level. Further research is needed on effective interventions and comprehensive models adapted for this care level.

## Background

Involuntary admission of adults to psychiatric hospitals has obvious implications for people’s autonomy and liberty, is ethically and professionally controversial and expensive for services and the wider society [1]. The use of involuntary admissions can also be traumatic for both the person in question and their relatives [1]. The United Nations Convention of the Rights of Persons with Disabilities from 2006 severely restricts the use of involuntary care towards persons with severe mental illness (SMI), and clarify their human rights to liberty, autonomy and free choice in matters of health and treatment [2].

In Norway, as well as internationally, reducing involuntary admissions within mental health care has been on the policy agenda for many years [3–6]. Despite the policy to reduce involuntary admissions, rates in many countries have increased or been close to constant over the last decades [7]. The rates vary greatly between countries. For instance, in 2015 Italy and Austria reported rates of 14.5 and 282 per 100 K people, respectively [7]. Norway reports numbers at the high end compared to other Western countries [8] with a rate of 186 per 100 K person 16 years and older in 2018 [9].

Very few studies have investigated interventions to reduce involuntary admissions. A systematic review only found 13 eligible RCTs [10]. The meta-analysis found that only advance statements, including advance directives and crisis plans, contributed to reduction of involuntary admissions. This is supported by other studies [1, 11, 12], although evidence is somewhat mixed [13, 14]. Other studies report that self-management [12], having alternatives additional to hospital services [15], contact with multiple services [16] and regular outpatient contacts [17] are associated with reduced involuntary admissions. Evaluations of the Trieste Model, where psychiatric hospitals were replaced by a network of Community Mental Health Centres that applied a framework of personal recovery and social inclusion, found that its effectiveness in keeping the rate of involuntary admissions low was achieved through a “whole system” approach, rather than the effectiveness of individual interventions [18].

Persons with SMI often use services from both primary and secondary health care level. Typically, acute mental health crisis services and involuntary admissions and treatment are the responsibility of secondary mental health care level, while primary mental health care provide non-acute services and general support [19]. This means that mental health professionals at the primary care level are frequently in contact with adults with SMI at a point when relapse occurs and therefore in position of detecting early signs. Primary health professionals also serves as gatekeepers to involuntary admissions, as most referrals come from General Practitioners (GPs) or out of hours medical emergency services [20]. This suggests that intervention at the primary health care level might facilitate or prevent pathways leading towards involuntary admissions. So far, research on service development aimed to reduce involuntary admissions has almost exclusively been conducted at the secondary level, and research on primary health care level is lacking. Of 121 studies identified in a broad scoping review concerned with efforts to prevent and reduce multiple forms of coercion and compulsion in the mental health context, only 10 were referred to under the category of community-based strategies, and most were from services provided at secondary health care level [19]. A review of studies focusing on involuntary admissions with data from Norway included 74 articles, none of which examined factors within primary mental health services or ways in which these might affect pathways to involuntary admissions [8]. There are also few qualitative studies on how to reduce involuntary admissions in the literature [19]. Strategies to reduce involuntary admissions involve practices in complex contexts. Detailed information on current practice, including factors contributing to or preventing involuntary admissions, is needed to investigate the potential for primary mental health services to impact, or reduce, involuntary admissions. To address this gap in the literature, the aim of this study was to explore mental health professionals’ experiences with factors within primary mental health services that might increase the risk of involuntary psychiatric admissions of adults, and their views on how such admissions might be avoided.

## Methods

Given the limited research in this field, we conducted a descriptive qualitative study, exploring research participants’ experiences and views through semi-structured individual interviews.

### Study setting

The current study was the first stage of a larger project that will develop and test, through a cluster randomized controlled trial, a primary care level intervention to reduce involuntary admissions (ClinicalTrials.gov, NCT03989765). The intervention arm consists of five Norwegian municipalities, in which the present interview study was conducted.

Norway has a publicly funded health care system, where primary mental health services are funded through the municipalities and secondary mental health services through the regional health trusts.

In Norway, involuntary psychiatric admissions are regulated in the Norwegian Mental Health Care Act §§3-2 and 3-3 [21], and they are effectuated at the secondary care level. Secondary mental health care is delivered through Regional Psychiatric Hospitals and their Community Mental Health Centres that provide community-based inpatient and outpatient treatment, including ambulant treatment. National health policies and directives to reduce the use of coercion has appointed the responsibility to reduce the use of involuntary psychiatric admissions to the secondary care level [22]. In the following, ‘involuntary admissions’ refers to involuntary psychiatric admissions of adults at the secondary mental health care level.

The 356 Norwegian municipalities are responsible for meeting its populations’ primary care needs. Primary mental health care often provides services to persons with SMI over long periods of time, commonly for years. Services provided can include everything from sheltered housing, day-care facilities, and therapeutic conversations, to helping with practical tasks in the house, transport to doctor’s appointments, handling medication, and assisting with leisure activities. GPs and out of hours medical emergency services are provided at the primary health care level. In addition, municipalities provide social care, (un)employment services and housing services. Many municipalities, including the five intervention municipalities, operate with a purchaser-provider split for allocation of their services, and this includes primary mental health services. A municipal Purchaser Office makes assessments based on individual needs, and issue decision letters that specify what services and extent of support an individual should get from primary mental health care.

### Participants and recruitment

Research participants were recruited from the five intervention municipalities. Eligible participants were professionals currently working within the municipality’s primary mental health services and who had supported someone who had been involuntary admitted. They were strategically recruited to get informants representing both managers and staff, and a variety of primary mental health services like sheltered housing, ambulant services, and activity/day care centres. Potential participants were identified by service managers in each municipality.

### Data collection

Data was collected through semi-structured individual interviews. The interview guide was structured around two overarching themes; (1) exploring pathways to involuntary admissions within current practice, and (2) exploring potential to prevent involuntary admissions. Interviewees were asked to describe one or two of the last incidents where they had been involved, directly or indirectly, in which someone was referred for involuntary admission. Managers were in addition asked some contextual questions on service organization, resources, and numbers of staff. The interview guides were developed by IW and TLH in collaboration with the research group of the larger project, which includes a peer researcher. IW and TLH carried out the interviews. In the first municipality they conducted them jointly, after which reflections on the functionality of the interview guide lead to no revisions. The rest of the interviews were performed separately by IW and TLH in two municipalities each. The interviews were carried out in meeting rooms at the municipality’s town hall or the relevant service’s location. The interviews lasted between 35 and 69 min, were audio recorded and transcribed verbatim.

### Data analysis

**Fig. 1:**
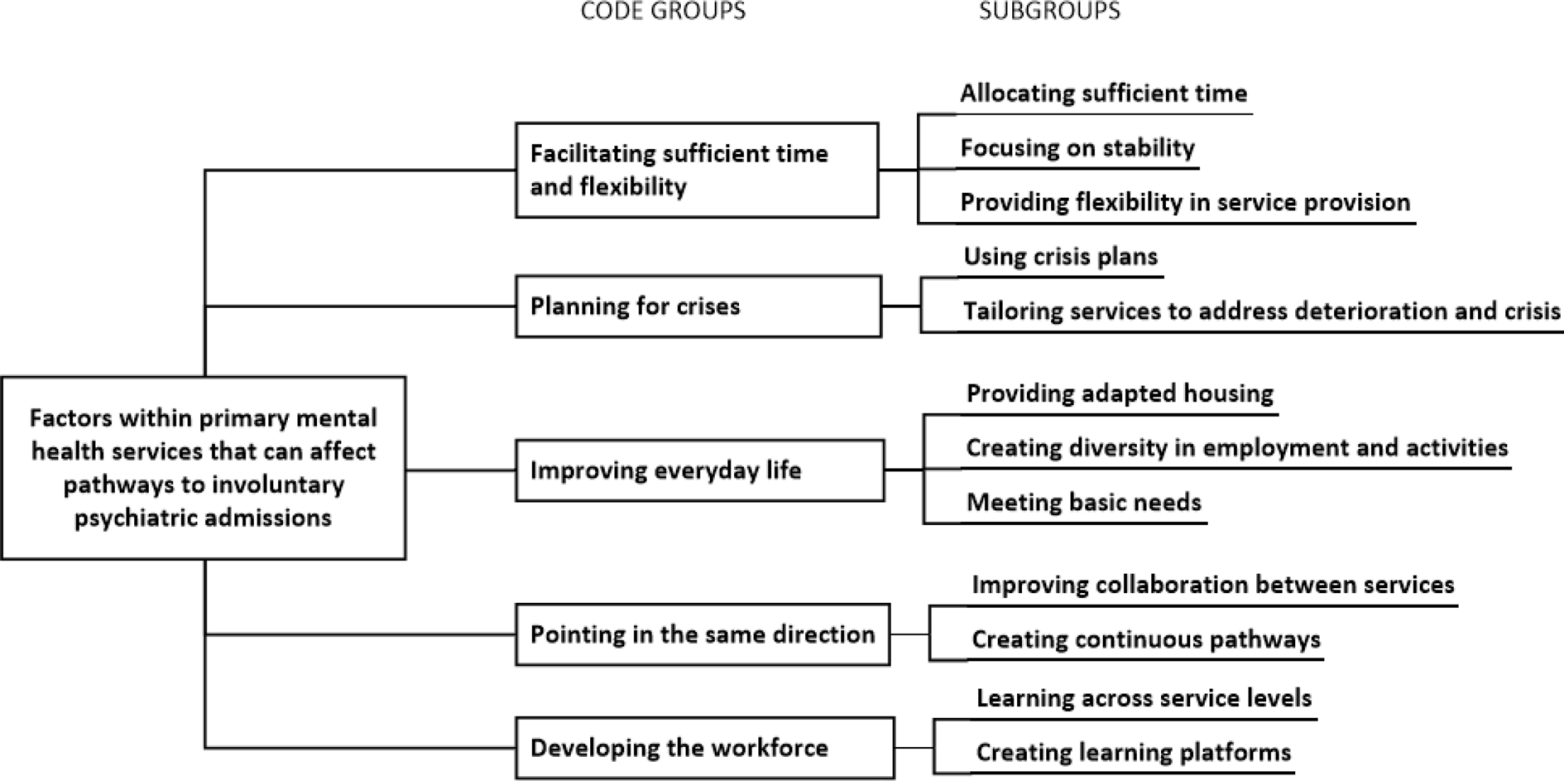
Final analytical code groups and subgroups

In line with the descriptive and explorative design of the study, data analysis followed the Systematic Text Condensation method [23, 24]. Analysis was performed in four steps. First, all transcripts were read in full by IW in order to form a total impression of the data material. Six preliminary themes were identified; (1) numbers unknown, (2) relationship, time, and stability, (3) individual adaption of activity, employment, and housing, (4) bureaucracy vs. flexibility, (5) collaboration with other services and (6) competence needed. The analytical software NVivo 12 pro was used in the second analytical step (QSR International). Here, IW systematically examined all transcripts and identified elements of text that elaborated the participants’ experiences of factors that could increase the risk of involuntary admissions and their suggestions of potential improvements that could facilitate a reduction. Malterud [24] calls such text fragments meaning units. On the basis of the preliminary themes the meaning units were coded and sorted. Corresponding codes within and across transcripts was gather into code groups. Thereafter, all authors read two randomly chosen transcripts and in agreement consolidated the code groups and their associated subgroups. Code groups were also reviewed within the context of previous research and theory. In the third analytical step IW represented the meaning content as written condensates, one for each subgroup. Participant quotes illustrating the meaning content of each subgroup were also identified. In the fourth analytical step, condensates and quotations were synthesized into an analytical description of the results. Results were written up by IW and the other authors contributed with reflections and critical revision. The final analytical code groups and subgroups are presented in Fig. 1. To complete step four, IW read eight randomly chosen original transcripts to assess whether the results reflected the data, which they did.

Below we illustrate our findings with direct quotations from participants. These were translated into English by the authors, and, to protect anonymity, are identified only with unique participant numbers, gender, and unique municipality numbers.

## Results

A total of 32 mental health professionals took part in the study, 14 managers and 18 staff from the five municipalities. Different primary mental health services were represented, including ambulant services, home care services, sheltered houses, and day care/activity centres. All the research participants had at least one year of experience working within mental health services, 23 had more than ten years’ experience. All but one participant had at least three years of higher professional training within health or social science. Information about the participants is described in Table 1.

**Table 1:** Description of the research participants

Variable	Informants (N = 32)	Percent
Sex		
Male	10	31.3
Female	22	68.7
Position		
Manager	14	43.8
Staff	18	56.2
Age group		
25–39	10	31.2
40–49	11	34.4
50–59	7	21.9
60–69	4	12.5
Level of education		
Vocational education training	1	3.1
3 years higher professional education	7	21.9
> 3 years higher professional education	24	75.0
Work experience within mental health services		
1–5 years	3	9.4
5–10 years	7	21.9
> 10 years	22	68.7
Work experience within present municipality		
< 1 year	5	15.6
1–5 years	8	25.0
5–10 years	4	12.5
> 10 years	15	46.9

Before describing the main results, two findings related to the study aim, form a backdrop. Firstly, the participants stated that their municipality in general provided good services to people with SMI. Secondly, during the interviews it became clear that while a few working in sheltered housing could recollect an estimated number of involuntary admissions within their service during the last year, none had the overview over the extent of involuntary admissions in their municipality nor the number of persons involuntarily admitted each year. This information forms an important part of the background on which to interpret the participant’s experiences.

The main results comprise the participants’ experiences with factors within current service provision that could increase the risk for involuntary psychiatric admissions, and their suggestions of how such admissions might be avoided. The results are presented according to the code groups as presented in Fig. 1.

### Facilitating sufficient time and flexibility

According to the participants, relationships and trust between them and the persons with SMI receiving their services were important to be able to prevent involuntary admissions. In their experience, there was no quick fix to establish a good relationship and trust with adults with SMI. Stability and sufficient time in their service provision were mentioned by the participants as important to prevent involuntary admissions. Over time this could potentially put them in a position to identify deterioration at an early stage, when easier to reverse. Many experienced that a factor in current practice that could increase the risk of involuntary admissions was lack of time. Budget cuts due to poor economy in the municipality, increased focus on efficiency and more people in need of help from primary mental health care, meant there was too little time to follow up each person, and made it harder to build good relationships and trust in the long run. One research participant described this as being caught in the “quantity vs. quality trap”. Political and health service management’s will to prioritize services towards this group of people was highlighted as a premise for sufficient resource allocation.

In addition, participants described the flexibility to act upon people’s shifting needs as important to be able to prevent involuntary admissions. In some municipalities the system for service allocation was mentioned as a factor that could increase the risk of involuntary admissions. This was highlighted by participants who experienced decisions made by the municipal Purchaser Office as rigidly controlling the amount of time they could spend with an individual. According to these participants, the service allocation hindered flexibility, left little time to act upon peoples shifting needs and were sometimes even contradictory to the participants’ professional assessment. That new measures to meet emerging needs could be delayed by the time it took for the Purchaser Office to process a request, was experienced to increase the risk of involuntary admissions. The participants who, when observing a person in deterioration, experienced having authority to spend some of their time more flexibly perceived this flexibility to be preventive.

“I’m not very happy about these decision letters [from the Purchasing Office]. […] if you’re supposed to be one hour here, one hour there, one hour there, then I think your work won’t get any focus. I’m not happy about not being able to get that. And there is something about, - in a way I do get it, that you need these decisions, but I just wish they weren’t so rigid about the hours.” (ID 51, female, municipality 5)

Another factor mentioned in some municipalities, was that lack of time and flexibility within their practice hindered assertive work towards people who did not yet have a decision for a particular service, but still had obvious needs. Some of the participants said they knew people who had SMI, but did not seek or want help, and who sometimes were involuntary admitted.

Some participants mentioned that more staff and resources were needed to get sufficient time to provide adequate care and facilitate flexibility in service provision. Others expressed that such improvements could be reached by prioritizing current resources within primary mental health care differently. Another potential improvement suggested by the participants was to organize allocation of service provision differently. A concrete suggestion was to categorize the decisions into “small”, “medium” and “large” amounts of time instead of specific number of hours. They argued that less rigid decisions could enable them to up- and down-scale service delivery according to a person’s present needs and thus give the flexibility in service provision needed to potentially prevent involuntary admissions. Allocating a proportion of time within the mental health services that the professionals could use in a flexible manner, was also mentioned as a possibility. Some argued that this could ensure that they had the time to work assertively to motivate persons without individual decisions to receive help.

### Planning for crises

Many participants described situations where people ended up in “revolving door patterns” of repeated involuntary admissions. Service development preventing such patterns were described as potentially contributing the most to reduce involuntary admissions. According to several of the participants, crisis plans could be a good measure to avoid this. In their experience, crisis plans made collaboration with the unwell person during crises easier, which could reduce the risk of involuntary admissions. Many of the participants experienced that a person’s stable periods were windows of opportunity to work together on an active, updated and functional crisis plan.

“And about coercion, I mean, that you get things imposed on you. Because many of ours [service users] have had kind of revolving door admissions, I mean in and out and in and out, over many years. And then there are those crisis plans, coping strategies. And I do see that we can prevent admissions too because we are involved. And I believe that’s important, at least for this group of users, because many of them have been admitted a lot and that is traumatic, I mean that is an additional burden. Simple as.” (ID 24, female, municipality 3)

The participants described great variation in how crisis plans were used, ranging from extensive, active and systematic use in some services, to others that hardly ever used such plans. In municipalities where the participants described no or more arbitrary use of crisis plans, this was expected to increase the risk of involuntary admissions. Different factors were mentioned including that services lacked routine to make, actively use and review crisis plans, that many persons declined when offered to make crisis plans, or that they said no to the measures in their crisis plan when they experienced a crisis.

Another factor within current practice mentioned by some was that crisis plans made by secondary health services during admissions were not always adapted to life outside the hospital setting. This was said to potentially increase the risk of repeated involuntary admissions. In some services, participants described that they used crisis plans more as the professionals’ plans for risk assessment. Such plans were made by staff without involving the person in question, and participants did not experience this use of crisis plans to have potential to reduce involuntary admissions.

Participants experienced it as sometimes difficult to get voluntary hospital admissions when someone was at an early stage of deterioration. They said that such referrals often were rejected, and that the situation had to be severe or even acute before a person got admitted, and at this point this frequently ended up as an involuntary admission. Some participants mentioned that in current practice the primary mental health care sometimes ended up passively watching further deterioration to the point where people got so unwell they got involuntary admitted, as the participants experienced the primary mental health services had no alternatives left that could help.

In line with the variation described in current practice, some participants indicated more potential than others in reducing involuntary admissions through improved use of crisis plans. Working out routines that secured structured and active use of crisis plans to all persons that could benefit from having one, was mentioned as a potential improvement measure. Another potential improvement mentioned was that better use of crisis plans could make GPs and out of hours emergency services aware of other options for persons in crises, and thereby prevent GPs and out of hours emergency services from assessing involuntary admission as the only option. Several participants suggested ways in which primary mental health care could reorganize services to be better able to prevent people from deteriorating to the point where involuntary admission became the solution. This included self-referral beds to persons in need of a short period of treatment, which could be established within or in connection with one of the housing facilities with round the clock staff. Other measures suggested was multidisciplinary teams working intensively towards persons during and after crises, increased availability of primary mental health care 24/7, and improved knowledge in GPs of what services the primary mental health care could offer persons when in crises.

### Improving everyday life

Making improvements to people’s everyday life was, according to the participants, one of the aims for primary mental health services. In some municipalities, the participants experienced lack of suitable housing alternatives for persons with SMI as a factor that could increase the risk of involuntary admissions. Narrow shelters, tight quarters in turbulent neighbourhoods, several persons with severe challenges living in the same place, limited protection against factors that could trigger relapses, and frequent drug use among neighbours, were described as environmental factors that made it difficult to de-escalate aggression or make worried persons feel safe. In one municipality participants had experienced that providing housing better adapted to a person’s circumstances and challenges had stopped her revolving door pattern of involuntary admissions.

“I do think there are cases where we can reduce the use of coercion too. Thinking of someone in supported housing for example […] So that too, differentiated accommodation, is extremely important, for some, to give them the opportunity to live on their own [i.e. independently] […] So that was important too, in a preventative view, as regards why that lady, for example [refers back to detailed discussion of accommodation tailored to individual needs], hasn’t [been involuntary admitted] as much as before. I am sure that had she lived at her previous accommodation there would’ve been so many trigger points that there would have been much, much more [involuntary admissions].” (ID 15, female, municipality 3)

Another factor mentioned in most of the municipalities was the lack of diversity in activities for persons with SMI. Lack of employment opportunities offered to persons with SMI were also mentioned.

“I think that if you’d managed to tailor support by means of activities… That’s why I think the collaboration that exists between Social Services and the municipality at the moment with IPS [Individual Placement and Support], individually tailored employment support, I think that’s a good entry, yeah, to prevent things. I believe so. At least for those at the starting line, I mean, who are about to build a mental disorder. That’s what I think. To get people to have self-worth, or to feel useful, you know. I think that is important. But, of course, there are those with severe disorders, where I also think that it’s about activity. And I’m saying that from what I see that some of them achieve, despite how really unwell they are.” (ID 30, male, municipality 2)

According to the participants, getting more diverse and adapted housing for people with SMI depended on this being a priority of the municipality’s housing services, as residential development is costly and demand resources. Some of the participants mentioned that providing the service model Individual Placement and Support could be an improvement to get persons with SMI employment. This was thought to potentially facilitate recovery and thus reduce involuntary admissions. In regard to activities the participants mentioned establishing more diversity in activities offered at the primary mental health care’s activity centres, better utilization of already existing activities, collaboration with voluntary organizations, increased use of human service assistants and transport assistance to go to activities, as potential measures to improve people’s possibility to engage in individual adapted activities. In municipalities where participants experienced that they offered a lot of activity opportunities, further improvements in diversity and individual adaption was still emphasized, as they still experienced people that did not engage in what they currently offered.

### Pointing in the same direction

Good collaboration between different services involved in an individual case was mentioned by the participants as important to be able to facilitate comprehensive treatment and coherence in service provision to persons with SMI. Collaboration with other services was, in many cases, seen as insufficient or lacking. Poor collaboration with GPs and secondary mental health services was considered to be a factor that could increase the risk of involuntary admissions. Collaboration with GPs was particularly emphasized, as they were the medical authority, within the primary health care level, on a person’s treatment and medication. The participants said that collaboration depended on the personal attitude and working preferences of the individual GP. They also mentioned that many GPs lacked knowledge about the services that the primary mental health care could offer. Collaboration with the secondary mental health services was also said to vary. Many participants mentioned that sometimes primary mental health care was not involved prior to a discharge and that this could result in people not getting adequate help as they returned home. Poor collaboration was described as potentially leading to lack of coordination between services from primary and secondary services, providing services in parallel rather that giving complementary support.

Another factor mentioned to affect collaboration between primary and secondary mental health care services in some municipalities, was that the primary and secondary service level held different professional perspectives. In the municipalities where this was mentioned to be a factor, the participants experienced that professionals at the secondary mental health services devalued the primary mental health care’s professional knowledge. This made it difficult to collaborate and agree on what was the right help to offer a person at transitions between care levels.

“Because the hospital and the specialist services often lecture us on what we should be doing and often have a shopping list for us when the service user gets out [of hospital]. And I think that specialist services, I mean, we see thing a bit different, they see the diagnosis and they see medication and they see treatment. And perhaps we see everyday opportunities more, and that you create a life, you must live life. You’re not supposed to be admitted and sort of not be, you’re not the illness, you know, you are something other than that. So, we have a bit different view on what is necessary when they get out.” (ID 2, male, municipality 1)

A general improvement mentioned by the participants was the primary mental health care’s collaboration with other services. Regular collaboration meetings between the primary and secondary mental health services, drawing up better routines for collaboration, and working up collaboration relations with staff in other services, were examples of suggested measures of potential improvement at an organizational level. At an individual level, collaboration meetings before discharge, deciding which service is responsible for what, and collaborating to make crisis plans, were some of the measures mentioned to improve collaboration between the services in their direct contact with the persons. Further, a joint understanding where both primary mental health care’s psychosocial recovery orientation and secondary mental health care’s more medical orientation was integrated in comprehensive service provision to persons with SMI, was mentioned as something to strive for. Some suggested establishing integrated multidisciplinary teams with staff from both service levels as a new measure that potentially could reduce involuntary admissions. A specific service model suggested was Flexible Assertive Community Treatment.

### Developing the workforce

Many participants experienced that a lack of competence to prevent involuntary admissions within their services could increase the risk of such admissions. A few of the participants mentioned that, particularly in supported housing services for people with SMI, some of the staff did not have relevant training. Some participants who had prior experience of working in acute wards in secondary mental health care said that employees there were trained to have the specialist competence needed to prevent crises and aggression from escalating. In their current practice within primary mental health care, on the other hand, they experienced a lack of such competence being provided to employees.

“What I feel I need is supervision and training courses and more competency, more spot on […] I have an impression that maybe it can be a bit like that, that if we all are better drilled, trained, supervised, that there will be more confidence and […] I think that could have an effect.” (ID 32, female, municipality 2)

Several of the participants mentioned that mental health professionals in primary mental health services should get more relevant knowledge on special areas related to this target group. Which potential improvements the participants described varied, both between the participants, the different services, and between the municipalities, but included knowledge on legislation regulating compulsion, evaluation of a person’s capacity to give informed consent, risk assessment, assessment of suicide risk, de-escalation techniques, personal recovery as framework for service provision, psychosis, tools to handle aggression, and medication. Here they suggested different measures to enhance the competence within the primary mental health services. Formal education or training courses, thematic tuition at internal staff meetings, staff reflection groups, and guidance from professionals form secondary mental health care level, including feedback on current practice were among the suggested measures. Joint professional development among staff from different services, exchange programs across service levels, and more guidance from secondary to primary mental health services, was also suggested.

## Discussion

Participants’ starting point was the primary mental health care in their municipality provided good services to people with SMI. Nevertheless, they described a number of factors that, in their experience, could increase the risk of involuntary admissions. Insufficient time and flexibility in long-term follow-up, limited resources, lack of or arbitrary use of crisis plans, lack of tailored housing, few employment opportunities, little diversity in activities offered, limited opportunities for voluntary admissions, inadequate collaboration between services, and lack of competence are examples of factors the participants mentioned. They suggested several improvements that potentially could be implemented to facilitate a reduction of involuntary admissions. Another finding was that none of the mental health professionals participating in the current study knew the extent of involuntary admissions of adults in their municipality.

### Putting involuntary admissions on the agenda

Our results showed that many participants experienced organisational factors that challenged prevention of involuntary admissions. Budget cuts, increased focus on efficiency, repeated reorganizations and rigid allocation systems for service provision were said to affect essential factors like stability, continuity, relationship, sufficient time to care and flexibility in their follow-up of persons with SMI. This is remarkably similar to what is reported from secondary care. Belling et al. [25] found that inadequate staffing levels, financial pressure, time pressure, heavy caseloads and models of decision making could give less patient contact time, more user discharge and have negative effect on continuity. This is also supported by other studies, where higher continuity of care in outpatient setting were associated with fewer hospitalizations and improved health outcomes for persons with SMI [26, 27]. Puntis et al. [28] argue that services need to focus both on continuity and flexibility because regular contacts (linear continuity) might fail to meet the fluctuating needs of those with SMI. Given these findings from secondary health care level, it might be reasonable to assume that organisational improvements facilitating factors like relationship, stability, continuity, sufficient time and flexibility in the primary mental health care’s long-term follow-up of persons with SMI, can potentially contribute to reduce involuntary admissions. For primary care to contribute in this regard the reduction of involuntary admissions needs to be put on the agenda and prioritised in the development of primary mental health care. None of the participants in our study, including the managers, were aware of the level of involuntary admissions within their municipality. This might imply that reducing involuntary admissions has not been systematically addressed, at least not within these municipalities. To monitor the numbers will allow the primary mental health care to evaluate questions like; Who are the persons that become involuntarily admitted in our municipality; Where do they live; and What mental health services do they receive. Likewise, they will be able to detect patterns of high use of involuntary admissions and endeavour to change them.

### Preventing revolving door patterns

In some municipalities and services, the participants experienced a lack of planning for crises as a factor that could increase the risk of involuntary admissions when crises appeared. Although the participants experienced crisis plans as an effective measure to reduce involuntary admissions, the use of such plans varied greatly. The potential to prevent revolving door patterns was emphasized in the participants’ suggestion to improve the use of crisis plans. According to Claassen and Priebe [29] revolving door patterns is a phenomenon that have followed the deinstitutionalization of mental health care. Recent reviews have found that crisis plans is one of the few measures shown to reduce involuntary (re)admissions [1, 11, 12], although the evidence is mixed as Thornicroft et al. [13] found no effect. As most existing research was conducted at secondary health care level, further research is needed to assess the effectiveness of crisis plans within primary mental health care. Although a crisis plan is a tool for crisis management it must, according to the participants, be prepared and actively addressed when the person for whom it is for is stable. This implies that one needs to take a long-term view, allowing sufficient time and stability in follow-up. This relates back to the organizational factors mentioned above: to be able to improve the use of crisis plans it might be necessary to address factors that can lead to insufficient time, stability, and flexibility in long-term care provision.

### Facilitating inclusion and personal recovery

The participants in our study experienced that lack of appropriate housing, employment, and activity opportunities for persons with SMI could increase the risk of involuntary admissions. That such everyday life contexts are of importance to personal recovery for those with SMI, is well acknowledged [30]. Ådnanes et al. [26] found that unmet needs for activity centre/day centre, meeting places, social services, and individual support contact, have negative effect on quality of life. Furthermore, recovery oriented treatment models like Housing First and Individual Placement and Support that provides help with housing and employment concurrently with mental health treatment, have demonstrated a positive effect on personal recovery and quality of life [31, 32]. Moreover, the tendency in European countries to create new ‘hospital-like’ living contexts within communities, clustering persons with SMI in the same facility and joint activities, has been criticized [29]. It can lead to exclusion and stigmatization and impede personal recovery [29]. Providing all those with SMI with adapted solutions within housing, employment and activities, requires access to a great variety of such facilities in the municipalities. This goes beyond the remit of primary mental health care and would involve reorganisation and redistribution of budgets in other municipal services such as social care, housing office, land planning, voluntary organizations offering activities and public and private workplaces. Thus, a call for reducing involuntary admissions needs to be put on the agenda across multiple sectors within the primary service level.

### Joining efforts across care levels

Persons with SMI often receive services from both primary and secondary mental health care, and health professionals at both level may share many of the same views and experiences regarding how best to provide support to avoid involuntary admissions. Collaboration across care levels might thus be a particularly important aspect as it could be of benefit not only to those using services but also to bot levels of care. In some municipalities in the current study, participants experienced that a complicating factor to such collaboration was that primary and secondary mental health services had a different perspective stemming from their different responsibilities and clinical focus. The participants experienced a knowledge hierarchy between primary and secondary mental health services, secondary care considering themselves as being on top. Participants suggested that combining resources and competency in a complementary manner could be a helpful way forward. Primary mental health participants could benefit from professional guidance from the secondary mental health care on specific issues regarding the target group, and better integration of the perspectives of primary care into secondary care could facilitate service provision, at both levels, better adapted to prevent involuntary admissions in individual cases.

As shown above, the participants experienced that the level of current collaboration with secondary mental health services varied. Lack of or poor collaboration was mentioned as a factor that potentially could be a risk for people ending up with involuntary admissions. Previous studies in Norway confirm that lack of collaboration between health services can lead to fragmented service provision and discontinuity in care [33]. One can thus assume that measures to improve collaboration across care levels can facilitate continuity in care and better coherence in service provision. Collaboration can also lead to joint efforts from mental health services at both care levels to provide voluntary alternatives, and thus facilitate a reduction of involuntary admissions. The participants described how services sometimes were provided in parallel rather than complementary support, showing that many of the themes from the results in the current study may be common to both primary and secondary mental health care level. Integrated multidisciplinary teams with staff from both service levels was suggested to avoid this. Flexible Assertive Community Treatment teams, which in a recent Norwegian evaluation showed reduction in involuntary admissions [34], was specifically mentioned.

Funding mechanisms and allocation of resources affect mental health service provision and thus might be factors affecting collaboration between care levels [35]. For instance, when health services are funded through fee-for-service, collaborative work might be given a lower priority because it does not release fees and thus represent a disadvantage to business [35]. With public funding of mental health services at primary and secondary care level, like in the setting of this study, the experience of limited funding and resources might give a ‘push’ effect where services try to disclaim responsibility in the follow up of persons with SMI instead of establishing a collaborative fellowship across care levels. Counteracting such effects is important to prevent persons with SMI ending up without the support they need from either care level.

Furthermore, if collaboration across care levels is good, reduction of involuntary admissions can be put on the agenda within both primary and secondary mental health care. National strategies and actions-plans place the responsibility to reduce involuntary admissions at the secondary health care level [4, 22]. The facilitation of such a reduction is expected to be done in partnership with the primary health care level. Improving the collaboration between care levels can thus help facilitate increased contributions from the primary mental health care level to the policy aim of reducing involuntary admissions.

### Strengths and limitations

The current study was limited to exploring mental health professionals’ experiences. Persons receiving primary mental health services, family and network, other services and relevant stakeholders might have different experiences. That managers in the municipalities held a “gatekeeper” role identifying potential participants could imply a bias in research participants. However, the service managers’ overview of the different services was helpful to identify eligible potential participants according to the recruitment strategy. This provided a sample with representation of both managers and staff, from a variety of services, and with many years of experience from primary mental health care. This gave data material characterized by thorough experience with current practice. All authors participated in the analysing process. This secured different perspectives and strengthened the results. The study was conducted in five Norwegian municipalities and the results are not necessarily generalizable to other contexts. Norway is a welfare state where health care is provided through well-developed publicly funded services. In this context the participants’ experiences are influenced by their expectations of such a health system, including long term follow up from health services. Results might have been different in other settings where health care services are structured or funded differently. Mental health services being organised in different care levels might have affected the participants’ views on factors affecting collaboration across services. Other factors might appear in contexts where mental health services are differently organised. Nevertheless, the fact that Norway face many of the same issues as other countries when it comes to involuntary admissions [8] implies relevance across contexts. The results were, with a few distinctions, recognizable across the included municipalities. Thus, it is likely that the experiences of participants in the current study is recognizable to other professionals working within similar services.

### Implications for practice

Based on the current study, there seems to be potential for primary mental health services to prevent some involuntary admissions. As such, for national policy to reduce involuntary admissions to be successful, the topic should find its place on the agenda of primary mental health care and form part of future service development at this service level. Specifically, primary mental health care should assess the need to improve the use of crisis plans and facilitate greater diversity in recovery-oriented service provision like housing, employment, and activities. In addition, competence to facilitate prevention of involuntary admissions should be improved at this service level. Further research is needed to explore other stakeholders’ perspectives, including persons with lived experience and their families. Furthermore, strategies adapted for primary mental health care level should be developed and tested to find effective measures for this care level. Finally, health authorities should to a greater extent incorporate primary mental health care in directives and incentives intended to reduce involuntary admissions.

## Conclusions

Professionals in primary mental health care experienced multiple factors in their service delivery and organization that could increase the risk of involuntary admissions. This could suggest that service improvements at this level potentially can facilitate a reduction of involuntary psychiatric admissions. Involuntary admission is the end-product of a process starting outside the hospital, implying this is where one should intervene when aiming to reduce use of involuntary admissions. The policy aimed to reduce involuntary admissions should therefore include primary mental health care service development. Continuity in service provision to adults at risk of involuntary admissions, diversity in recovery-oriented measures like housing, employment and activities for people with SMI, collaboration with other services provided to adults with SMI, and competence on prevention of involuntary admissions, needs to be prioritized within primary mental health care. Health authorities’ incentives to reduce involuntary admissions should incorporate the primary mental health care level to a greater extent. Further research is needed on effective interventions and comprehensive models aimed at reducing involuntary admissions adapted for this care level.

## Data Availability

The datasets generated and analysed during the current study are not publicly available.

## Abbreviations

SMI: Severe mental illness
GP: General practitioner
